# Cumulative risk assessment methodology applied to non-dietary exposures: developmental alterations in professional agricultural settings

**DOI:** 10.1186/s12995-025-00451-y

**Published:** 2025-02-28

**Authors:** Luca Tosti, Martina Marazzini, Mohammed Kanadil, Francesca Metruccio

**Affiliations:** 1 Department of Biomedical and Clinical Sciences, University of Milan, International Centre for Pesticides and Health Risk Prevention ASST Fatebenefratelli Sacco, Milan, Italy; 2 Department of Biomedical and Clinical Sciences, University of Milan, Milan, Italy; 3 International Centre for Pesticides and Health Risk Prevention, ASST Fatebenefratelli Sacco, Milan, Italy

**Keywords:** Pesticides, Non-dietary exposure, Occupational, Mixtures, Cumulative risk assessment

## Abstract

The safety assessment of combined exposure to xenobiotics has been an emerging topic for several years. Methodologies and approaches for cumulative risk assessment (CRA) are being developed primarily for the dietary risk assessment of pesticides, thus focusing only on consumer exposure. However, as highlighted in recent years, non-dietary exposures, such as those encountered by professionals in agricultural settings, may pose a significant risk due to their higher levels compared to dietary exposure. Despite this, existing methodologies for cumulative risk assessment (CRA) have not adequately addressed this critical aspect. In the EU, even if several legislations highlight the need to address the non-dietary CRA, no legal requirements are in place yet, therefore, there are no harmonized methodologies and approaches currently available. In a first step to fill this gap, this study is aimed at exploring CRA methodology applied to non-dietary exposure to pesticide in agricultural settings, specifically targeting operators, re-entry workers, and bystanders. The primary objective of the study was to verify the feasibility of an electronic register of plant protection treatments as data source for identifying and characterizing in field mixtures and consequently estimating cumulative non-dietary systemic exposure in real life. The relevant active substances selected for this investigation were those listed in foetal craniofacial alterations cumulative assessment groups (CAGs), established by the European Food Safety Authority (EFSA) for dietary CRA. Exposures to pesticides were estimated using European Union agreed mathematical models. The preliminary findings of this investigation effectively revealed the value of the register of treatments in assessing real-life plant protection products (PPP) usage in professional agricultural settings. In conclusion, the study provides encouraging insights into using the pesticide dietary CRA approach for non-dietary risk assessment in agricultural settings, underlining the necessity for further research and investigation of its feasibility for the evaluation of other acute effects but also for long-term effects related CAGs.

## Background

Pesticides are crucial for modern farming, enabling effective pest control and enhancing crop productivity world-wide. However, concerns regarding their potential adverse effects on human health and the environment led to continuous improvement in monitoring technique together with revised regulation and pesticides pre-marketing risk assessment procedures around the world.

Pre-marketing process in the risk assessment of pesticides represents an essential step to ensure the safety of plant protection products (PPP) aimed at identifying and evaluating the potential risks for human health and the environment associated with the use of a new pesticide before it is placed on the market. This assessment, inherently complex, encompasses a wide range of aspects, ranging from the toxicity of the active ingredient and its metabolites/degradation products to environmental and human health impacts, as well as its persistence and ability to accumulate in soils and in plant and animal organisms.

Nevertheless, it is crucial to note that the current EU pesticide assessment evaluates risk based on individual substances, focusing on one pesticide at a time. Recently, the European Commission has provided non-binding guidance to consider the cumulative effects of substance mixtures on the same biological target, thereby aligning assessments more closely with real-world exposure scenarios and potential human health risks. While various regulatory EU authorities and frameworks recognize the significance of assessing health risks associated with combined exposure to multiple substances, there is a lack of harmonized methodologies.

For instance, Regulation No. 1907/2006 (REACH) [1] addresses aggregate exposure^1^ for consumers but does not specify risks associated with combined chemicals. Similarly, the Classification, Labeling, and Packaging (CLP) Regulation No. 1271/2008 [2] establishes criteria for classifying marketed mixtures without considering the combined effects of individual substances, although it does highlight the need to evaluate synergistic and antagonistic effects without providing a specific methodology. Currently, there is no standardized approach for non-dietary risk assessments of pesticides; instead, methodologies used for biocidal products [3] are applied by pesticide regulatory assessors. These methodologies primarily focus on intentional mixtures—those containing multiple active substances—or aggregate exposure. The guidance for biocidal products regulation (BPR) [3] includes a tiered approach for assessing human health risks from combined exposures, employing hazard indexes based on the nature and effects of involved substances. The first tier evaluates each substance individually without accounting for combined effects to identify those that may pose risks based solely on their individual toxicity. The second tier sums the risk matrices of each substance to assess the overall mixture risk while still not considering combined effects. The third tier focuses on substances targeting common organs or systems, providing a more accurate assessment by incorporating specific reference values and safety factors. A similar tiered approach is suggested for cumulative exposure assessments, utilizing simpler deterministic models at lower tiers and more complex probabilistic models at higher tiers.

In contrast, both the United States and Canada have established legal requirements for conducting cumulative risk assessments of intentional and incidental mixtures across various legislative contexts (pesticides and other substances). Detailed methodologies for non-dietary cumulative exposure assessments are outlined in specific guidance documents integrated into regulatory processes [4–9]. In the U.S., pesticide non-dietary cumulative risk assessments for residential exposures are conducted for substances within common mechanism groups (CMGs), which include compounds with similar chemical structures and modes of action, such as organophosphates and synthetic pyrethroids. However, these methodologies have not been specifically tailored to address occupational cumulative risk assessments for pesticides.

At EU level, methodologies and approaches for cumulative risk assessment (CRA) are primarily being developed for the dietary risk assessment of pesticides, focusing on consumer exposure, thanks to the availability of consumption and residue monitoring data [10].

In 2009, for such purpose, the Panel on Plant Protection Products and Their Residues (PPR Panel) and EFSA collaborated to develop a procedure for establishing Cumulative Assessment Groups (CAGs) for pesticides [11], grounded in their toxicological profiles. When this methodology was applied to acute and chronic effects of the nervous system, it identified five groups of CAGs [12], while according to EFSA the application on chronic thyroid effects led to two CAGs [13, 14]. More recently, two CAGs were added, pertaining craniofacial developmental alterations of foetuses: craniofacial alterations due to abnormal skeletal development (CAG-DAC) and head soft tissue alterations and brain neural tube defects (CAG-DAH) [15].

Craniofacial alterations encompass a range of birth defects impacting skull and facial development, varying in severity and significantly affecting a child's health and well-being. While the precise causes remain unclear, exposure to specific pesticides has been associated with higher risks for these developmental defects in laboratory animals. The mechanisms through which pesticides induce craniofacial alterations are not completely elucidated, though they may disrupt neural tube development and interfere with biological processes crucial for foetal growth [16].

Foetal developmental effects are critically relevant to occupational reproductive health, particularly for pregnant employees, as they can disrupt vital stages of fetal growth and lead to long-term health consequences. The Council Directive 92/85/EEC [17] emphasizes the importance of protecting female workers during pregnancy and maternity by mandating employers to assess exposure to specific chemical agents that may pose reproductive risks. This includes a focus on substances known to induce reproductive toxicity, which encompasses both developmental toxicity before and after birth. The cumulative risk assessments (CRA) for developmental alterations considered in the present study is particularly pertinent for women of childbearing age, especially among agricultural operators and re-entry workers, who may be exposed to harmful chemicals during early-stage critical developmental windows, at which time female workers are often unaware of their pregnancy.

Given that over 400 active substances are approved in the EU [18], it is reasonable to expect that these compounds are often mixed in actual agricultural practices. This work was aimed at exploring the application of the dietary CRA methodology to the non-dietary exposure of agricultural operators, re-entry workers, and bystanders, who are generally exposed to significantly higher dose levels compared to consumers.

For this purpose, the CAG of substances eliciting craniofacial alterations has been selected to perform CRA related to reproductive health of professional operators and workers as well as bystander and residents.

As mixture identification and characterization plays a pivotal role in CRA process, the present work explored the usefulness of a regional (Lombardy – Italy) PPP treatment register, the "Quaderno di Campagna" (QdC), to identify and characterize mixture events in agricultural settings.

As mentioned before, several subjects were taken into consideration for their potential mixture exposure; these include agriculture operators (individuals responsible for applying pesticides in agricultural settings), re-entry workers (individuals returning to treated areas shortly after pesticide application for various tasks such as harvesting or maintenance), and bystanders (individuals present near pesticide application sites but not directly involved in the process of application). Residents, who are individuals living adjacent to an area where pesticides are applied, were not assessed, as their acute exposure is considered covered by bystander exposure scenario.

In the EU, non-dietary exposure to pesticides is typically estimated using appropriate exposure models and is conducted according to the intendent use of the PPP reflected in the labels, e.g. crop type, growth stage, application method and amount of active ingredient. In the initial tiers of non-dietary risk assessment, mathematical models—primarily deterministic—are employed based on conservative assumptions and default values. This approach often leads to precautionary worst-case scenario assessments, resulting in a substantial margin of safety. For instance, these models typically incorporate default parameters such as the hectares of treated areas for specific crops and task-related factors influencing professional exposure.

In this context, utilizing data from the "Quaderno di Campagna" (QdC) database offers several advantages. It allows for the extraction of time-resolved applied doses and the extent of treated areas for each specific crop, reflecting actual usage data rather than relying solely on default values used in initial tier assessments.

## Methods

### “Quaderno di Campagna” (QdC)

To comply with the record-keeping requirements set by the Directive on Sustainable Use of pesticides (Directive 2009/128/EC), a register of PPPs treatments was established in Italy (DPR n.55/2012), more commonly known as the “Quaderno di Campagna” (QdC). In 2014 the Lombardy Region (Regional Phytosanitary Service—RPS) developed and made available to all Farmers an electronic register of PPPs treatments to monitor and improve the efficiency of pesticide use.

Farmers are required to fill out the QdC with detailed information on each PPP treatment, including the type of product used, the application rate, the treatment date, and other relevant information.

The QdC database, last updated on June 26th, 2023, contains 385,017 treatments recorded over seven years (2016 to 2022), from two distinct sources named “CSV” (derived from the file format, comma-separated values) and “SisCO” (the Farms’ Portal of the Lombardy Region). Since data from the “CSV” source were submitted to the RPS by means of third parties’ software/platforms they were more prone for potential typing or conversion errors, particularly affecting data related to treated areas, thereby compromising data consistency. Given the high level of uncertainties in correcting and cleaning this partially unreliable data and considering the exploratory nature of the present work, it was deemed more appropriate to solely analyse data from the SisCO source, whose data are directly entered into the online database through the farms’ portal of the Lombardy Region and are therefore subject to former checks by the competent authority.

PESTIDOC [19] and the Ministry of Health's Plant Protection Products Database served as sources for retrieving information such as the amount (in grams) of active substances in individual PPPs, details from labels (formulation type, number of treatments per year, pesticide use and application volumes), withdrawal dates, etc.

### Identification of the mixture event in the database

The first step to identify mixture events was to retrieve PPPs containing the active ingredients belonging to CAG-DAC and CAG-DAH in the QdC. The reference list of these two groups of active substances was retrieved in the EFSA’s Guidance “Retrospective cumulative dietary risk assessment of craniofacial alterations by residue of pesticides” [15].

As only commercial name of PPPs is recoded in the QdC, the PESTIDOC database was additionally consulted to link commercial products and active substances. Ultimately, the following information was cross-referenced to identify the mixture event: company ID, operator, date, type of treatment, and commercial product (combined with its active substance or substances).

As the considered developmental defects are acute effects, PPP mixtures application scenarios scouted were those that could occur in a single working day.

Relevant PPPs mixture events were those satisfying the following combination of conditions (Table 1).

**Table 1:** PPPs’ mixture application types

Tank mix application	Consecutive application	Formulated mixture
Same farmer	Same farmer	Application of a PPP containing more than one active substance grouped in the same CAG
Same day	Same day	
Same hectares treated	Different hectares treated	

### Estimation of exposure

In occupational settings, exposure to pesticides occurs mainly via dermal during normal handling of concentrates, application of diluted products, in case of accidental spilling in case of operators, or dried foliar residues in case of re-entry workers. Additionally, exposure can also occur via inhalation during mixing and loading of concentrated products (vapours), during application of diluted products (spray drift) and volatilization of deposited residues (Table 2). Considering the current outdoor scenarios, inhalation exposure was considered relevant for operators and bystanders only.

**Table 2:** Routs and factors of exposure

	Operator	Re-entry worker	Bystander
**Route**			
**Dermal**	During mixing, loading, and application	Post-application, during re-entry activities (e.g.: weeding, harvesting)	During application, through drifted particles
**Inhalation**	During or after pesticides application by inhalation of particulates	Exposure through vapours (negligeable in outdoor)	During or after pesticides application by inhalation of drifted particles
**Exposure factors**			
	• Product formulation type • Crop type^a^ • Application rate^a^ • Water volume applied^a^ • Area treated	• Crop type^a^ • Application rate^a^ • Exposure duration • Dislodgeable Foliar Residue (DFR) • Dermal transfer Coefficient (TC) • Number of applications^a^	• Application rate^a^ • Spray drift • Area treated

^a^Input values from QdC

Exposure values were estimated for each active compound of the selected mixture event. For the operator, total potential dermal and inhalation exposure during mixing, loading and application were estimated using an in-house model based on the Agricultural Operator Exposure Model (AOEM) [20] considering relevant exposure factors such as product formulation type, crops treated, application rates, water volume applied and treated area (Table 2). The data provided by SisCO unfortunately does not indicate the number of operators involved simultaneously in the same treatment/field. Consequently, the number of hectares treated per entry have been set to the maximum value specified in the calculation model AOEM. This has partially prevented overestimation of assigning treated hectares by multiple operators to a single operator. For worker re-entering treated crops, the total potential dermal exposure was estimated according to the Europoem II model [21].

Crop type, application rate, exposure duration, dislodgeable foliar residue (DFR) and crop specific dermal transfer coefficient (TC) were considered as relevant exposure factors for re-entering activities. Additionally, since multiple applications can lead to accumulation of residue on crop leaves, the maximum number of applications reported in the product label was further considered to estimate dermal exposure as a worst case (Table 2). Re-entry worker inhalation exposure was not estimated as it was considered negligeable in the outdoor scenario.

Operator and worker exposure estimation include protection factors of protective equipment if they were indicated in product label.

For bystanders, total potential dermal and inhalation exposure to spray drift was estimated using the methodology described by Martin et al. 2008 [22], considering the default adult total body surface area of 1,66 m^2^ and applying reduction factor of 18% for light clothing [23]. Relevant exposure parameters considered were the application rate, percent of drift (amount of off target spray cloud- crop specific), area treated and exposure duration (Table 2). Typically, risk assessment of bystanders includes exposure evaluation of adult and child, however, given the health effect under investigation, the risk assessment of child was not conducted.

### Dermal and inhalation absorption

When conducting pesticide risk assessment active substances dermal absorption value is needed to obtain systemic exposure (internal dose) from estimated potential exposure (external dose). Due to the complexity, variability and specificity of dermal absorption rates among products, as well as limited availability of experimental data, default values (25%-10% for concentrate organic-based and water-based products, respectively, and 70%-50% for diluted organic-based and water-based products, respectively) [24] were considered for systemic exposure estimation.

Moreover, given the exploratory nature of the present work, the highest default DA values, for operators, were considered (25% for concentrate and 70% for diluted).

Based on a preliminary analysis comparing estimated systemic exposures using default and experimentally derived DA values for selected PPPs, no substantial differences were noted. This was attributed to the use of personal protective equipment (PPE) protection factors that lower systemic exposure by approximately 90%. Since almost all labels of relevant mixture products indicated the use of PPE, this significantly influenced the systemic exposure estimate, overshadowing the differences between the default and experimental DA values.

Re-entry workers’ and bystanders’ exposure assessment are currently reliant on default exposure parameters (crop specific- transfer coefficient, -dislodgeable foliar residue and -drift values) which possess a considerable level of uncertainty that generally leads to unrealistically high and conservative exposure estimates.

In light of the above, to take into consideration that using a higher DA value would enhance further the exposure estimation value, it was decided to consider (for re-entry workers and bystanders) also the lower DA default to counterbalance the addition of too many conservative worst case default values.

This approach allows to obtain upper (worst case) and lower (best case) bound of the exposure estimates range and consequently a better understanding of the supposed real exposure (Table 3).

**Table 3:** Re-entry worker best- and worst-case parameters assessed

	Best case	Worst case
**Dermal absorption %**^**a**^	10	70
**P (Clothing protection factor)**^**b**^	0.9 (= 90%)	1 (= 100%)

^a^Re-entry worker and bystander

^b^Re-entry worker

For re-entry worker in particular, analogous reasoning has been applied to clothing penetration factor considering best case scenario with a 10% penetration through clothes and worst case scenario considering an almost naked worker (Table 3): (i) a best-case scenario in which the active ingredient is poorly absorbed through the epidermis and clothing provide a protection factor of 90%: (ii) a worst case scenario, where the active ingredient is mostly completely absorbed and no protection from clothing.

Inhalation absorption was assumed to be 100% for operators and bystanders.

### Cumulative risk assessment

Cumulative risk assessment evaluates potential risk that could result from systemic exposure to multiple chemicals with common mechanisms of toxicity or similar adverse effects. The grouping of these chemicals, known as cumulative assessment groups (CAGs), is established using expert judgment and scientific criteria based on dose addition model which is widely accepted by global regulatory and non-regulatory scientific bodies like the US EPA, WHO, and EFSA for decision-making in the absence of specific mixture data [25].

The next step is the hazard characterization, which implies the evaluation of the toxicity and dose–response relationships used to derive specific no effect levels for each pesticide within the CAGs.

Following hazard characterization, a cumulative exposure assessment is performed. This step estimates the combined exposure to multiple pesticides within each CAG using agreed modelling to assess the distribution of cumulative exposure.

Thereafter, in order to evaluate the cumulative risks from systemic exposure to multiple chemicals that act via similar toxicological mechanisms or have similar adverse health effects, the Total Margin of Exposure (MOET) is used as a metric for cumulative risk assessment. Before delving into the Total Margin of Exposure, it's essential to outline the basic concept of the Margin of Exposure (MOE). The MOE is a ratio that compares the no observed adverse effect level (NOAEL) or the benchmark dose (BMD) of a substance to the estimated human exposure level (Eq. 1).

1 $$MOE=\frac{NOAEL\;or\;BMD}{Estimated\;Human\;Exposure}$$

A higher MOE indicates a greater safety margin. Regulatory agencies often use MOE values to make risk management decisions, with lower MOE values indicating a possible concern and a potential need for regulatory actions.

The MOET provides a measure of the safety margin between the mixture systemic exposure and the level at which adverse effects are not expected. The approach used for the derivation of a combined Margin of Exposure (MOET) consisted in calculating the reciprocal of the sum of the reciprocals of individual margins of exposure (MOEs) to each chemical contributing to the risk (Eq. 2). This approach is extensively detailed in the work of More et al., 2019 [25].

2 $$MOET = {\left(MO{E}_{1}^{-1 }+ MO{E}_{2}^{-1 }\dots +MO{E}_{n}^{-1}\right)}^{-1} = {\left(\frac{{E}_{1}}{Rf{P}_{1}}+\frac{{E}_{2}}{Rf{P}_{2}} \dots + \frac{{E}_{i}}{Rf{P}_{n}}\right)}^{-1}$$

RfP*n* is the toxicological reference point (i.e., NOAEL) for chemical *n* and E*n* its exposure.

For MOET, the RfP*n* refers to the specific NOAEL for craniofacial alteration for each active ingredient as identified in the work of Anagnostopoulos et al., 2022 [15]. For human safety assessment a MOET above 100 is considered protective by the European Commission Standing Committee on Plants, Animals, Food and Feed (SCoPAFF).

## Results

### Characterization of pesticide mixtures

The total QdC database consisted of 4481 treatment entries with 602 different plant protection commercial products. Among these, 98 were formulated with active ingredients included in CAG-DAC and 105 in CAG-DAH, for a total of 203 commercial products. The analysis of pesticide mixture events revealed a diverse range of combinations of active ingredients.

**Fig. 1:**
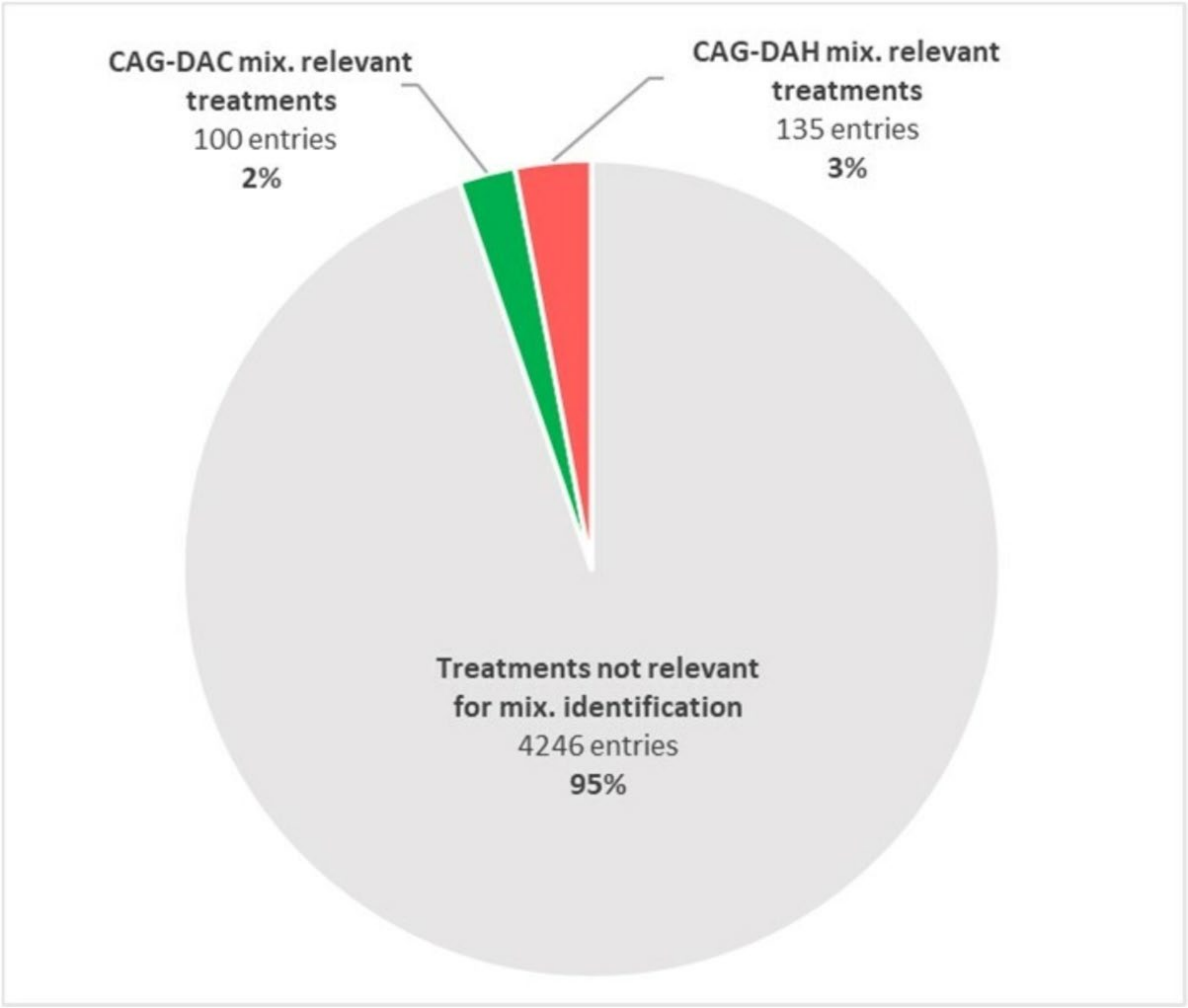
Mixture events identification for CAG-DAC and CAG-DAH

The identification of mixture events across the database was carried out considering tank mix events, consecutive treatments and formulated mixtures (refer to Table 1). Results showed that 2.2% and 3.0% of treatments encompass mixtures of active substances belonging to DAC and DAH CAGs, respectively (Fig. 1).

**Fig. 2:**
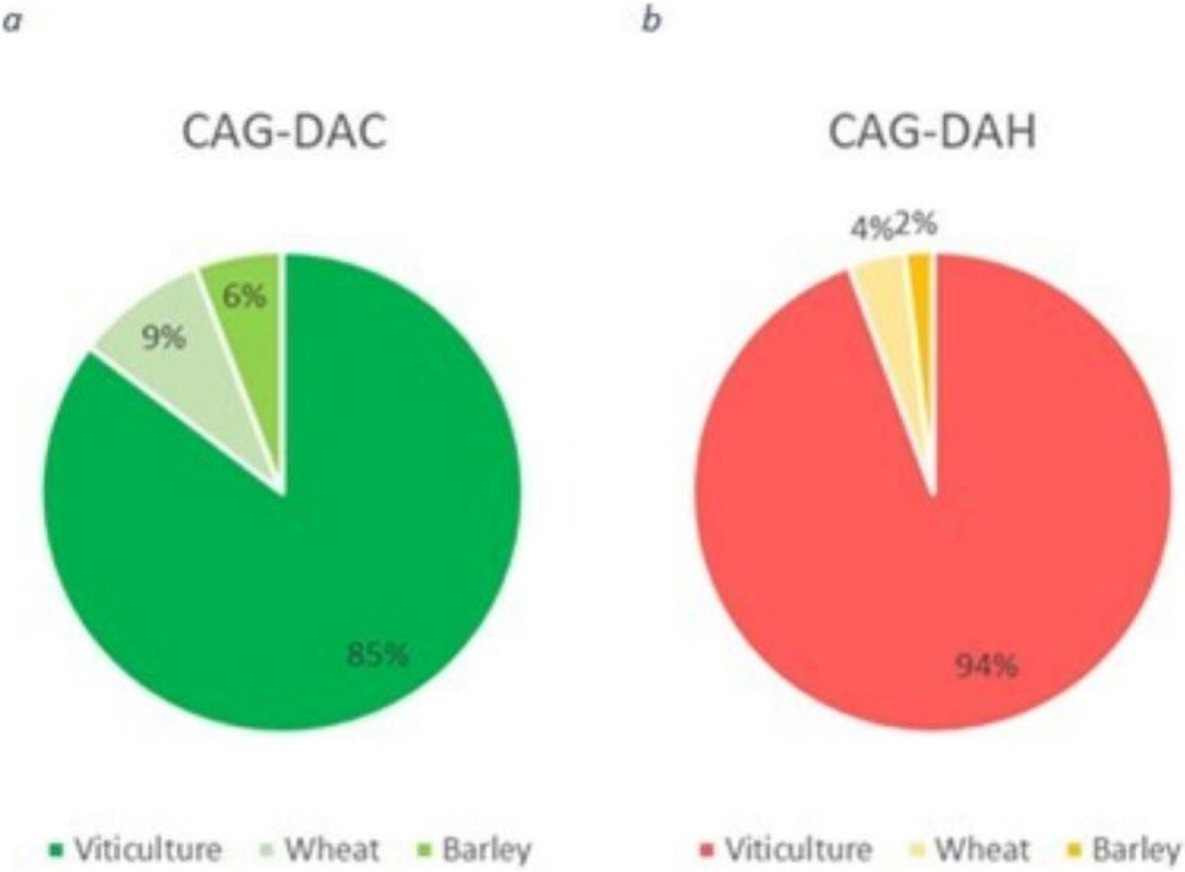
Mixture events distribution by crop (**a**) CAG-DAC and (**b**) CAG-DAH

In relation to the CAG-DAC, the QdC database reports a total of 34 mixture events by 15 agricultural operators, representing approximately 15% of the entire cohort of farmers recorded in the QDC dataset. A substantial proportion (29 out of the 34) of recorded mixtures, were specifically employed in viticultural, thus encompassing roughly 85% of the total DAC relevant mixture events observed (Fig. 2a).

With respect to CAG-DAH, the QdC database documented 53 mixture events carried out by 18 farmers out of 99 farmers recorded in the QDC database. The analysis revealed that 50 of these mixtures, accounting for 94% of the total mixture events, were utilized in viticulture (Fig. 2b).

**Fig. 3:**
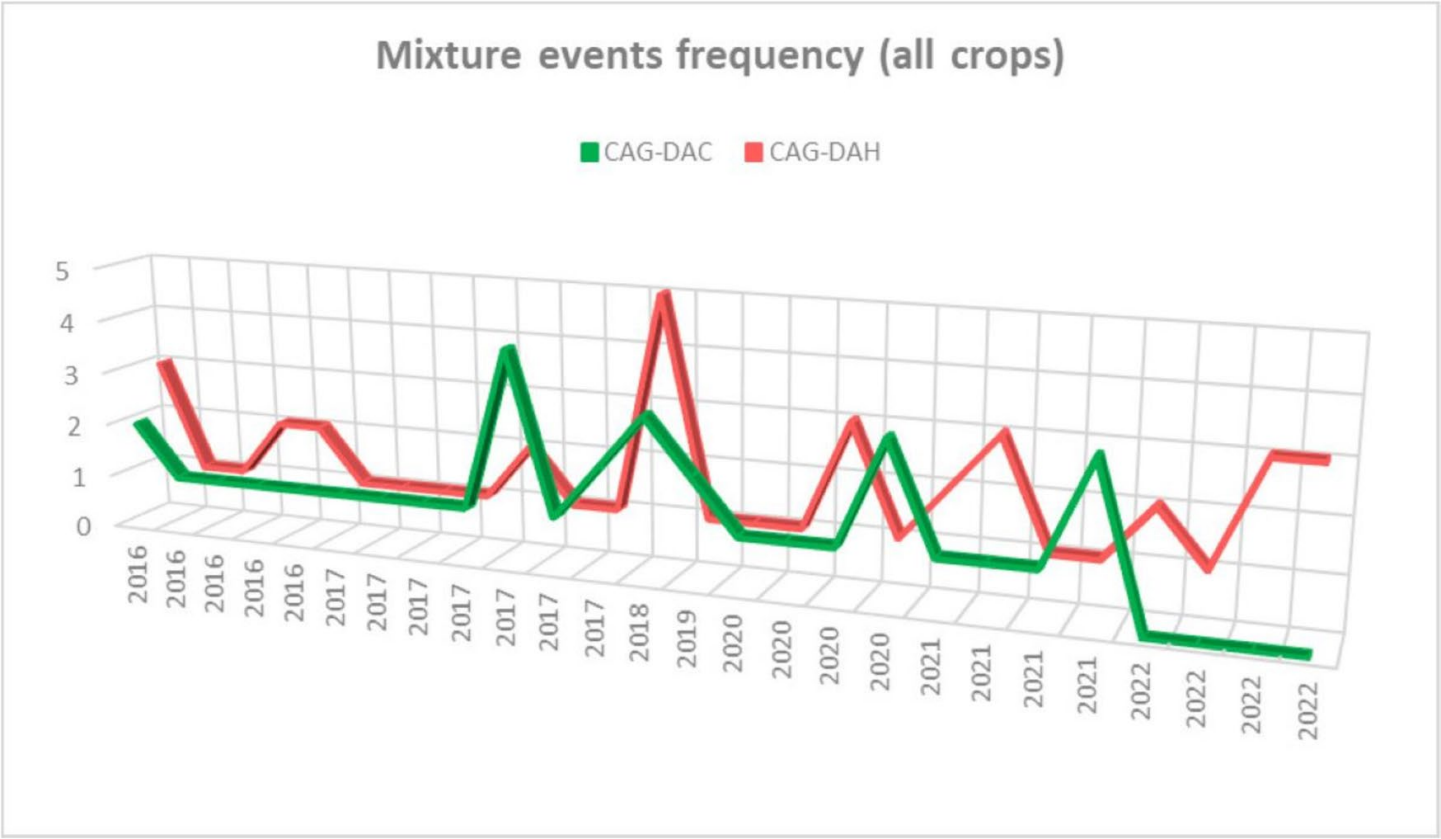
Mixture events frequency (all crops)

The frequency of use for these specific mixtures was low: 4 times a year for DAC and 5 times a year for DAH (Fig. 3) as a maximum in 2017 and 2018 respectively.

Following a thorough examination, it emerged that nearly every mixture event involving substance in CAG-DAH also involved CAG-DAC as a number of active compounds belong to both craniofacial alteration CAGs. In light of that and considering that the virtual totality of mixture events is on viticulture, it was decided to focus the analysis on CAG-DAC active ingredients relevant mixture events on grapes only.

For the concerned mixtures, 13 active ingredients were identified and characterized, based on their specific toxicity reference values (RfP) for MOET (Table 4).

**Table 4:** RfPs of investigated active ingredients

Active ingredient	NOAEL CAG-DAC (mg/kg bw)^a^
Abamectin	0.8
Cymoxanil	25
Cyproconazole	12
2,4-D	5
Deltamethrin	10
Epoxiconazole	60
Fenpyrazamine	300
Folpet	1^b^
Mancozeb	10
Metconazole	12
Prothioconazole	2
Spiroxamine	30
Tebuconazole	1^b^

^a^From EFSA 2022[15]

^b^NOEALs derived from LOAEL divided by 10

### Exposure

Systemic exposure estimation to pesticide mixtures in operators, re-entry workers, and bystanders is summarized in Table 5. Values of exposures were obtained from the AOEM model for the operators, EUROPOEM II model for re-entry workers, and the methodology of Martin et al., 2008 for bystander. Application rates for these active ingredients vary, with folpet and spiroxamine appearing frequently across different mixtures. Operators, who directly handle and apply the pesticides, tend to have lower estimated exposure levels compared to re-entry workers. For instance, in mixture 2 with folpet (AR 1.48 kg/ha), operators have a total estimated exposure of 0.021 mg/kg bw per day, while re-entry workers have significantly higher exposure levels (2.45 and 17.16 mg/kg bw per day for best case and worst-case scenario, respectively). In viticulture, re-entry workers generally experience high estimated exposure levels due to specific viticulture scenario characterized by intense and extensive contact with the canopy. Additionally, another factor contributing to higher re-entry worker exposure is the total residue accumulation from multiple applications throughout the year.

**Table 5:** Operators, re-entry workers and bystanders’ single substance estimated exposure data (mg/kg bw per day)

ID mixture^a^	A.I	AR A.I. kg/ha	Operator		RE-entry worker		Bystander	
			**DA (%)**	**TOT. EXP**	**BC**^**b**^	**WC**^**c**^	**BC**^**b**^	**WC**^**c**^
2	FENPYRAZAMINE	0.98	10/50	0.012	0.40	2.77	0.00355	0.02814
2	FOLPET	1.48	10/50	0.021	2.45	17.16	0.00536	0.04250
4	FOLPET	2.39	10/50	0.041	0.97	6.76	0.00865	0.06862
4	SPIROXAMINE	0.75	25/70	0.013	0.73	5.09	0.00272	0.02154
6	FOLPET	5.29	10/50	0.129	5.56	38.90	0.01916	0.15189
6	SPIROXAMINE	2.05	25/70	0.017	1.99	13.91	0.00742	0.05886
7	FOLPET	4.77	10/50	0.035	5.01	35.07	0.01727	0.13696
7	SPIROXAMINE	2.46	25/70	0.016	2.39	16.70	0.00891	0.07063
8	FOLPET	0.60	10/50	0.031	0.63	4.41	0.00218	0.01731
8	SPIROXAMINE	0.35	25/70	0.020	0.34	2.38	0.00127	0.01006
9	FOLPET	0.60	10/50	0.031	0.63	4.41	0.00218	0.01731
9	SPIROXAMINE	0.35	25/70	0.020	0.34	2.38	0.00127	0.01006
10	FOLPET	0.65	10/50	0.033	0.68	4.78	0.00235	0.01861
10	SPIROXAMINE	0.39	25/70	0.022	0.38	2.65	0.00140	0.01107
11	MANCOZEB	0.56	10/50	0.021	0.34	2.38	0.00201	0.01596
11	CYMOXANIL	0.06	10/50	0.002	0.08	0.54	0.00020	0.00158
11	FOLPET	0.55	10/50	0.019	0.71	4.98	0.00200	0.01584
12	FOLPET	0.74	10/50	0.027	0.72	5.02	0.00269	0.02130
12	CYMOXANIL	0.21	10/50	0.009	0.27	1.90	0.00076	0.00599
13	FOLPET	0.74	10/50	0.027	0.72	5.02	0.00269	0.02130
13	TEBUCONAZOLE	1.13	10/50	0.040	1.10	7.67	0.00411	0.03257
14	MANCOZEB	0.19	10/50	0.008	0.15	1.07	0.00067	0.00532
14	CYMOXANIL	0.06	10/50	0.044	0.06	0.41	0.00022	0.00172
14	FOLPET	0.40	10/50	0.059	0.39	2.71	0.00145	0.01149
15	FOLPET	0.59	10/50	0.068	0.62	4.34	0.00215	0.01702
15	TEBUCONAZOLE	0.04	25/70	0.001	0.04	0.27	0.00013	0.00102
15	SPIROXAMINE	0.20	25/70	0.007	0.19	1.36	0.00072	0.00567
16	FOLPET	0.74	10/50	0.027	0.72	5.02	0.00269	0.02130
16	TEBUCONAZOLE	1.13	10/50	0.040	1.10	7.67	0.00411	0.03257
17	TEBUCONAZOLE	0.05	25/70	0.001	0.05	0.34	0.00017	0.00136
17	SPIROXAMINE	0.17	25/70	0.004	0.16	1.15	0.00060	0.00479
18	FOLPET	1.13	10/50	0.039	0.46	3.20	0.00410	0.03253
18	TEBUCONAZOLE	0.09	10/50	0.003	0.09	0.61	0.00032	0.00253
19	FOLPET	1.18	10/50	0.033	0.48	3.34	0.00427	0.03385
19	TEBUCONAZOLE	0.01	10/50	0.0004	0.01	0.07	0.00002	0.00016
20	MANCOZEB	1.46	10/50	0.051	1.71	11.97	0.00528	0.04186
20	CYMOXANIL	0.02	10/50	0.038	0.01	0.06	0.00008	0.00065
21	MANCOZEB	1.16	10/50	0.088	1.12	7.87	0.00420	0.03326
21	TEBUCONAZOLE	0.09	10/50	0.004	0.07	0.48	0.00031	0.00244
22	MANCOZEB	1.03	10/50	0.087	1.00	6.99	0.00374	0.02963
22	TEBUCONAZOLE	0.48	10/50	0.010	0.47	3.26	0.00174	0.01378
23	FOLPET	0.84	10/50	0.007	0.98	6.89	0.00305	0.02419
23	SPIROXAMINE	0.26	25/70	0.001	0.25	1.76	0.00095	0.00756
24	FOLPET	1.05	10/50	0.007	1.23	8.61	0.00381	0.03024
24	SPIROXAMINE	0.38	25/70	0.002	0.37	2.58	0.00138	0.01096
25	SPIROXAMINE	0.20	25/70	0.001	0.19	1.36	0.00073	0.00578
25	FOLPET	0.72	10/50	0.006	0.70	4.89	0.00262	0.02079
28	FOLPET	2.72	10/50	0.018	5.93	41.54	0.00985	0.07813
28	SPIROXAMINE	0.26	25/70	0.006	0.25	1.76	0.00095	0.00757
29	CYMOXANIL	0.11	10/50	0.001	0.04	0.31	0.00038	0.00305
29	SPIROXAMINE	0.26	25/70	0.005	0.25	1.76	0.00095	0.00757
30	CYMOXANIL	0.11	10/50	0.001	0.04	0.31	0.00038	0.00305
30	ABAMECTINE	0.01	10/50	0.004	0.004	0.03	0.00002	0.00019
31	FOLPET	0.80	10/50	0.012	0.84	5.88	0.00290	0.02295
31	SPIROXAMINE	0.31	25/70	0.007	0.30	2.10	0.00111	0.00881
32	FOLPET	0.80	10/50	0.012	0.84	5.88	0.00290	0.02297
32	SPIROXAMINE	0.35	25/70	0.004	0.34	2.38	0.00127	0.01006
33	FOLPET	0.80	10/50	0.012	0.84	5.88	0.00290	0.02297
33	SPIROXAMINE	0.35	25/70	0.004	0.34	2.38	0.00127	0.01006
34	FOLPET	0.80	10/50	0.012	0.84	5.88	0.00290	0.02297
34	SPIROXAMINE	0.35	25/70	0.004	0.34	2.38	0.00127	0.01008

*A.I* Active ingredient, *AR* application rate, *DA* % of dermal absorption (concentrated/dilution), *BC* best case, *WC* worst case, *Tot. Exp.* total exposure includes dermal and inhalation exposure during mixing&loading, application

^a^ID mixture: refer to mixtures in grapes

^b^Dermal Absorption: 10%

^c^Dermal Absorption: 70%

Bystanders, who are nearby but not directly involved in the application process, have estimated exposure values significantly lower than re-entry workers, but comparable to operators for the worst case. In mixture 7 with folpet (AR 4.77 kg/ha), bystanders have estimated exposure levels of 0.017 mg/kg bw per day (best case) and 0.136 mg/kg bw per day (worst case), which, while significantly lower than re-entry worker exposure (5.01 mg/kg bw per day—best case), is comparable to that of the operators (0.035 mg/kg bw per day), in the best case.

Overall, folpet appears frequently and generally results in higher estimated exposure levels across all categories, especially at higher application rates. Mixture 28, with Folpet at an AR of 2.72 kg/ha, shows very high single substance estimated exposure levels for re-entry workers (5.93 mg/kg bw per day, best case and 41.54 mg/kg bw per day, worst case). On the other hand, spiroxamine, often paired with Folpet, shows instead lower single substance exposure.

Not surprisingly, the data indicates that re-entry workers are generally at the highest level of exposure, followed by operators and bystanders. This is expected for non-dietary exposure assessment in viticulture scenario for the reason described above.

### Cumulative risk assessment

**Fig. 4:**
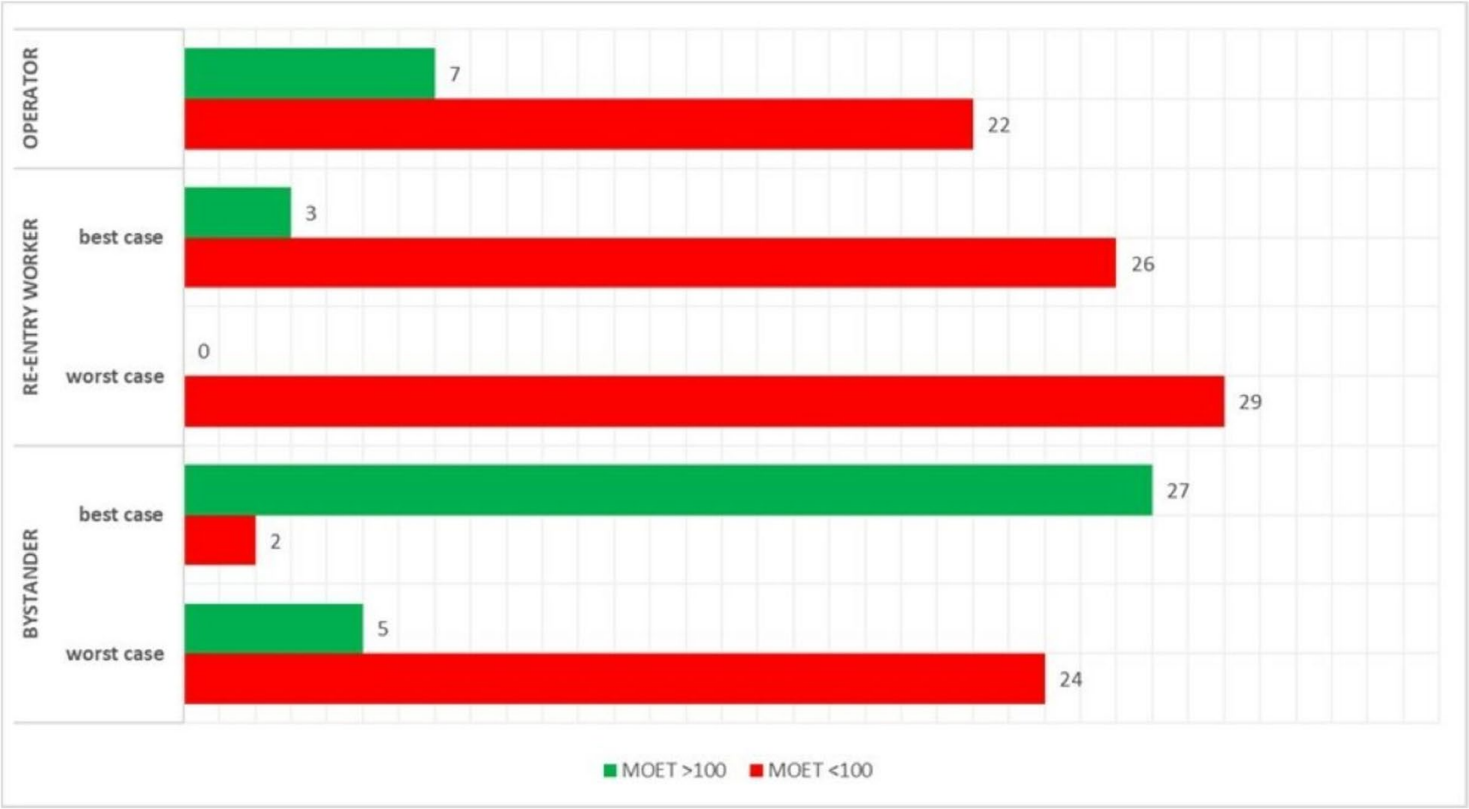
Risk assessment summary result

The assessment of cumulative risks for craniofacial alterations in operators, workers, and bystanders revealed a wide range of MOET across different exposed groups (Table 6 and Fig. 4).

**Table 6:** Operators, re-entry workers and bystanders cumulative risk assessment data

Id mixture^a^	Operator	Re-entry worker		Bystander	
		**BC**^**b**^	**WC**^**c**^	**BC**^**b**^	**WC**^**c**^
	**MOET**	**MOET**	**MOET**	**MOET**	**MOET**
2	48	4	< 0.1	186	23
4	24	10	0.1	114	14
6	8	2	< 0.1	52	6
7	28	2	< 0.1	57	7
8	31	16	0.2	449	57
9	31	16	0.2	449	57
10	29	14	0.2	418	53
11	48	13	4	453	57
12	37	14	0.2	368	46
13	15	6	< 0.1	147	19
14	16	25	8	656	83
15	14	15	0.2	435	55
16	15	6	< 0.1	147	19
17	693	185	3	5205	656
18	24	18	0.3	226	29
19	30	21	0.3	233	29
20	151	58	0.8	1883	237
21	76	55	0.8	1374	173
22	54	18	0.3	473	60
23	150	10	0.1	324	41
24	137	8	0.1	259	33
25	155	14	0.2	378	48
28	55	2	< 0.1	101	13
29	4625	982	14	21179	2671
30	199	1465	21	22396	2825
31	85	12	0.2	341	43
32	85	12	0.2	340	43
33	85	12	0.2	340	43
34	85	12	0.2	340	43

*MOET* total margin of exposure, *BC* best case, *WC* worst case

^a^ID mixture: refer to mixtures in grapes

^b^Dermal Absorption: 10%

cDermal Absorption: 70%

### Operators

Out of 29 mixtures, 7 mixtures are considered safe based on MOET. The remaining 22 mixtures fall below the safety threshold of 100, hence needing refinement. Among mixtures that resulted below 100, the mean, minimum and maximum values were 42,8 and 85, respectively. The lowest MOET value obtained was 8 (folpet-spiroxamine; mixture ID 6). Folpet had shown to be the active ingredient most involved in the mixture events having the lowest MOET values, followed by spiroxamine and tebuconazole.

### Re- entry workers

For the assessment of re-entry workers, who might work in treated areas post-application, exposure levels are generally higher in viticulture scenario.

In the best-case scenario in which the active ingredient is poorly absorbed through the epidermis and clothing provide protection, 3 out of 29 mixtures meet the MOET acceptability criteria. This indicates that the majority of the mixtures pose potential risks and require refinement. Among mixtures that resulted below 100, the mean, minimum and maximum values were 15, 2 and 58, respectively. The lowest MOET value obtained was 2, in a folpet-spiroxamine mixture. Folpet again showed a similar trend when it comes to the risk of mixtures, having the lowest MOE values, followed by spiroxamine and tebuconazole.

In the worst-case scenario, the estimation of cumulative risk falls below the threshold limits in all cases. This is considered an expected outcome since the assumptions for this scenario include maximum dermal absorption and the absence of protective clothing. In light of this and considering the results of the best-case scenario, it seems appropriate to focus solely on the best-case scenario.

### Bystanders

Bystanders, who are nearby but not directly involved in the application process, generally have significantly lower exposure than re-entry workers but can still be remarkable depending on the mixture and dermal absorption rate.

For these individuals, in the best-case scenario in which the active ingredient is poorly absorbed through the epidermis, two mixtures were marginally below the MOET safety threshold of 100. All other mixtures resulted to have a high MOET.

For the worst-case scenario characterized by a high dermal absorption, 24 mixtures were below the MOET safety threshold of 100. Among mixtures that resulted below 100, the mean, minimum and maximum values were 258,6 and 2825, respectively. The lowest MOET value obtained was 6, in a folpet-spiroxamine mixture. Similar to the previous outcomes, folpet was the active ingredient more frequently used in the mixture with the lowest MOETs.

### Risk drivers

From these results, it was clear that folpet was the active substance with a significant presence in mixtures with the lowest MOET values. Building on that, the need for investigating the individual role for each substance contributing the saturation of the MOET was deemed crucial.

**Fig. 5:**
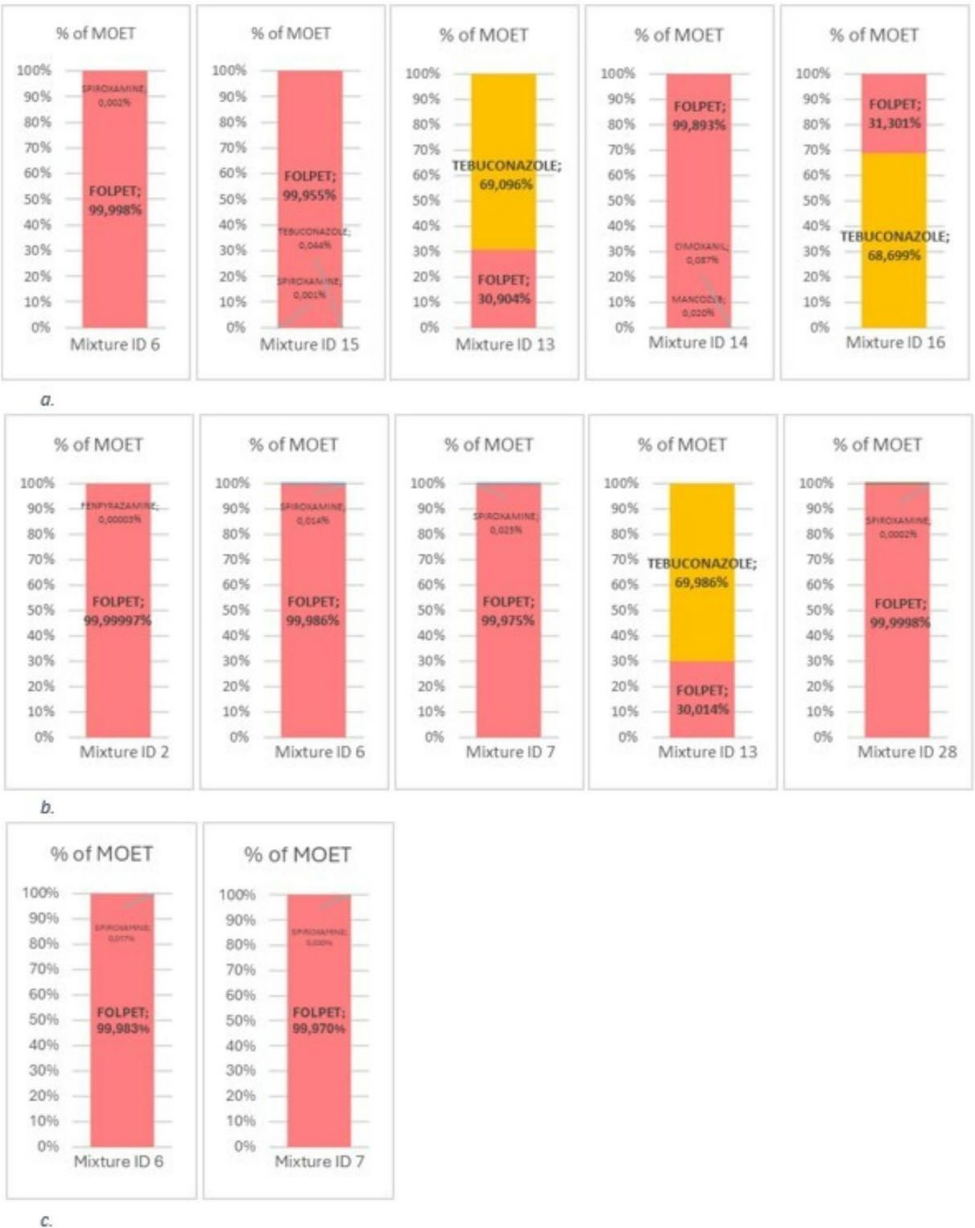
Risk driver contribution to selected mixtures. **a** Operator, **b** Re-entry worker, **c** Bystander

As part of the exposure assessment and subsequent calculation of MOETs (Tables 5 and 6), mixtures with the lowest MOET values where further investigated to scout the percent contribution of each active substance to the overall mixture MOET for operators, re-entry workers and bystanders (Fig. 5a, b, c). Considering the 5 mixture events with the lowest MOET for operators and re-entry workers, folpet is the active substance that most contributes to the risk metric in the majority of cases. For bystanders’ best case, folpet again resulted to drive the saturation of the risk metric in the two mixture events with a MOET lower than 100 (Fig. 5c). For all these individuals, when folpet is the risk driver, its contribution to the risk matric is virtually total (i.e. its individual MOE is less than 100). The reason for this dominant contribution is the result of its higher application rate and(or) a low specific NOAEL for craniofacial alterations compared to the other components of the mixtures.

On the other hand, when considering the two- and one-mixture events with tebuconazole as the active substance risk driver for operators and re-entry workers respectively, it appears that the application rate is the primary factor contributing to the risk matrix, as specific NOAELs for CAG-DAC/DAH of the active substances in the mixtures (tebuconazole and folpet) are equal.

## Discussion

Pesticides play a fundamental role in modern agriculture enabling effective pest control and enhancing crop productivity globally. However, their potential hazard on human health and the environment requires continuous improvements in pre-marketing risk assessment procedures. These assessments are complex, encompassing various factors such as the toxicity of active ingredients and their degradation products, environmental fate, and the occurrence of these substances in soils, water, living organisms and food supplies.

One significant aspect highlighted is the current limitation of risk assessments being conducted on single substances rather than considering the potential cumulative effects of multiple substances exposure. This approach does not accurately reflect real-life scenarios where individuals are often exposed to mixtures of pesticides.

Currently, as no legal requirements to address CRA are in place at EU level, no harmonized methodologies and frameworks are used by different regulatory silos.

In the last years, efforts have begun to address this issue by developing methodologies for CRA, but these have focused solely on dietary exposures in consumers. This was possible thanks to robust and up to date European diet consumption data that, together with harmonized residue levels monitoring system, allow consumer exposure estimation.

On the counterpart, no such level of information is available to characterize non-dietary exposure to pesticide mixtures, for both occupational and residential settings. Up to now, in fact, given that no EU requirement is in place to collect and monitor agricultural uses of plant protection products, data is collected only by some Member States in the remit of their national regulation and therefore hard to be retrieved.

Only recently, the European Commission adopted an implementation of regulation No. 1107/2009 regarding the content and format of records of PPP kept by professional users, that will enter into force only in 2026. As a result, methodologies or approaches to address non-dietary CRA are at the moment at the outset.

Fortunately, ahead of their time, the region of Lombardy, in Italy, has developed and implemented an electronic register of plant protection treatments (QdC) to comply with the record-keeping requirements set by the Directive on Sustainable Use of pesticides (Directive 2009/128/EC). These records were analysed to identify and characterize mixture events under real field conditions, challenging the cumulative risk assessment methodology for non-dietary systemic exposure related to CAGs on craniofacial alterations serving as a methodological exploratory pilot study.

The study's findings reveal that a minor proportion of the QdC pesticide treatments involve active ingredients associated with craniofacial developmental alterations (CAG-DAC and CAG-DAH). However, viticulture is identified as the major crop where these mixtures are employed, highlighting the need for targeted risk assessments in this sector. The estimated exposure levels for different individuals—operators, re-entry workers, and bystanders—vary significantly, with re-entry workers experiencing the highest exposure levels due to their intensive contact with treated plants and potential accumulation of residues from multiple applications per year.

Among the active substances used in pesticide mixtures, folpet is identified as the greatest contributor to the exposure estimates and risk levels across all individuals followed by tebuconazole. This is due to their high application rates together with low specific NOAELs (No Observed Adverse Effect Level) for craniofacial alterations.

The preliminary result of this research suggests a potential non dietary cumulative risk for acute human health effects (potentially triggered by a single exposure event), particularly for individuals with high exposure levels such as agricultural re-entry workers. Furthermore, given the nature of the effects, it is important to emphasize gender-specific vulnerabilities, especially among re-entry workers where childbearing age females representation is not neglectable, and among female bystanders whose presence is incidental and unrelated to work involving plant protection products.

This is particularly relevant when considering the specific CAG-DAC skeletal anomalies susceptibility window; experimental evidence showed that this sensitive window is between day 8 and day 10 of embryogenesis in rodents, which correspond to days 17–22 of pregnancy in humans. It is worth mentioning that in this period women are often unaware of their pregnancy and may not yet be following good pregnancy general guidelines related to chemical exposure [26, 27].

Nevertheless, it is important to point out that these results should be treated with cautions since several limitations, that can lead to an overestimation of cumulative exposure and risk, and limitations related to data collection from electronic registers need to be acknowledged and considered in this investigation.

As previously mentioned, the current analysis utilized data from the electronic register of the Lombardy region, as this was the only available database for the authors at this time. However, while pesticide usage is also recorded by other Italian regions, there is currently no national coordination in place. This lack of a centralized repository hinders comprehensive comparative analysis.

It is also important to note that in Lombardy, the compilation of the register and pesticide use (QdC) is mandatory for farms that exceed certain crop area thresholds. For all other smaller farms, QdC compilation is voluntary. As a result, the data sample may not be representative of the entire agricultural sector in Lombardy.

Further uncertainties are associated with exposure estimates and toxicological characterization of identified CAG-DAC risk drivers, folpet and tebuconazole.

For exposure estimates, uncertainties include mainly (i) incomplete data on application methods leading to incomplete accuracy of exposure characterization, (ii) unavailable data on the number of operators involved in the application process, which could impact (overestimation) exposure assessments, particularly in large treatment areas, (iii) the reliance on default dermal absorption values and the inherent overestimation in exposure estimates, and (iv) re-entry worker exposure default values such as Transfer Coefficient (TC) and Dislodgeable Foliar Residue (DFR) that are recognized to be particularly conservative and therefore are under revision following recommendations from EFSA [23]. In addition, re-entry worker exposure estimates are affected by worst case assumption on the maximum number of applications indicated in product labels and by the conservative default re-entry time after last application.

For the toxicity characterization of CAG-DAC risk drivers, folpet and tebuconazole, uncertainties pertaining the NOAEL setting were identified during the CRA of dietary exposure to pesticide residues conducted previously by EFSA. In particular, the specific NOAELs for DAC of these two active compounds could not be identified, as specific effects for the allocation in this CAG were observed at the LOAEL, therefore the NOAEL was set dividing the effect level by a factor of 10, leading to a likely overestimation of toxicological potency of the substances. Moreover, according to the uncertainty analysis performed by the EFSA [15], the availability of a toxicity study with a more adequate dose spacing would likely lead to a higher NOAEL, and consequently a higher MOET. In addition, a further uncertainty regarded the study design of folpet critical study, that raises the possibility of misclassifying skeletal variation with malformations. Therefore, the overall EFSA Weight of Evidence (WoE) assessment based also on the above uncertainties, concluded that the CAG-DAC membership probability of folpet is between 40–70%, while of tebuconazole is between 90–99% [15].

Moreover, it should be noted that for the substances folpet and tebuconazole, the NOAELs for craniofacial alterations were identified after the peer review assessment of these actives and are lower than the critical effect NOAELs used for establishing Health Based Guidance Values (HBGVs). This issue was managed by treating it as a significant uncertainty in the uncertainty analysis in the retrospective cumulative dietary risk assessment of craniofacial alterations due to pesticide residues.

Anyhow, a preliminary first-tier sensitivity analysis of our data performed only on mixtures with active substances for which a specific NOAEL was clearly identified (e.g., without folpet and/or tebuconazole), showed that one out of three mixtures had a MOET close to 100 for re-entry workers.

In essence, while the CRA outcomes of the present work are not yet mature enough to support risk assessment decisions, this study effectively demonstrated the value of the QdC in assessing real-world PPP usage and in identifying pesticide mixture events in professional agricultural settings. The QdC enabled the identification of mixture events for selected active substances, crops on which are used, their frequency of use, and their qualitative and quantitative compositions, providing a solid foundation for characterizing pesticide mixtures.

## Conclusions

This initial investigation, to evaluate the non-dietary cumulative risk in agriculture occupational settings, emphasizes the importance of evaluating pesticide mixtures besides individual substances to better characterize the cumulative risks posed by these chemicals.

This investigation also identified several limitations, including incomplete datasets and conservative assumptions, which primarily affect risk characterization. Nevertheless, this preliminary study provides valuable insights into the identification and characterization of non-dietary systemic exposure to pesticide mixtures, demonstrating the usefulness of a record-keeping PPP treatment register.

The purpose of this work is primarily to illustrate a methodological approach. This approach, having been applied to a very small sample (pilot study), should be utilized by other authors in different contexts and exposure scenarios, such as on vines, orchards, and cereals in various agricultural settings, as well as on other cumulative risk assessment groups (CAG) for both acute and chronic assessments. In the near future, this can be achieved thanks to the EC adoption of implementation No. 2023/564 of regulation No. 1107/2009 regarding the content and format of records of PPP kept by professional users. This implementation will enter into force and application in 2026, and therefore, could generate a substantial amount of EU representative PPPs real-use data sufficient for consideration by the competent authority to potentially implement the non-dietary cumulative risk assessment (CRA) within the legislative framework.

## Data Availability

No datasets were generated or analysed during the current study.

## Footnotes

1 Total exposure an individual has to that substance from multiple sources and through various pathways.
